# The Scale Effects of Organometal Halide Perovskites

**DOI:** 10.3390/nano13222935

**Published:** 2023-11-13

**Authors:** Yibo Zhang, Zhenze Zhao, Zhe Liu, Aiwei Tang

**Affiliations:** 1 Key Laboratory of Luminescence and Optical Information, School of Physical Science and Engineering, Beijing Jiaotong University, Ministry of Education, Beijing 100044, China; 2 School of Chemistry, Food and Pharmacy, University of Reading, Reading RGE 6AH, UK; la804577@student.reading.ac.uk; 3 Beijing Engineering Research Center of Mixed Reality and Advanced Display, School of Optics and Photonics, Beijing Institute of Technology, Beijing 100081, China; 3220215084@bit.edu.cn

**Keywords:** halide perovskites, scale effects, carrier diffusion, excitonic properties, defects

## Abstract

Organometal halide perovskites have achieved great success in solution-processed photovoltaics. The explorations quickly expanded into other optoelectronic applications, including light-emitting diodes, lasers, and photodetectors. An in-depth analysis of the special scale effects is essential to understand the working mechanisms of devices and optimize the materials towards an enhanced performance. Generally speaking, organometal halide perovskites can be classified in two ways. By controlling the morphological dimensionality, 2D perovskite nanoplatelets, 1D perovskite nanowires, and 0D perovskite quantum dots have been studied. Using appropriate organic and inorganic components, low-dimensional organic–inorganic metal halide hybrids with 2D, quasi-2D, 1D, and 0D structures at the molecular level have been developed and studied. This provides opportunities to investigate the scale-dependent properties. Here, we present the progress on the characteristics of scale effects in organometal halide perovskites in these two classifications, with a focus on carrier diffusion, excitonic features, and defect properties.

## 1. Introduction

Since 2009, organometal halide perovskites (OHPs) have emerged as facile solution-processed semiconductors for photovoltaic (PV) applications [1,2,3,4]. Beyond high-performance PV devices, other optoelectronic devices such as light-emitting diodes (LEDs) [5,6,7,8,9,10], lasers [11,12,13,14,15,16], photodetectors [17,18,19], solar cells [9,20], and transistors [21] employing these materials have also been achieved with promising prospects. The in-depth understanding of the fundamental properties of hybrid perovskites is a must. It can further serve as a guiding principle in the experimental designs to meet the requirements of materials under various conditions.

Polycrystalline films of OHPs are usually employed for photovoltaics and photodetectors. It is now expected that the OHP single crystals with much lower trap densities can exceed the performance of the polycrystalline films. Filterless narrow-band photodetectors have been demonstrated using solution-processed polycrystalline films with full width at half-maxima (FWHM) below 100 nm [18]. The FWHM of the photodetectors can be further shrunk to <20 nm using single-crystal OHPs as the active layer [19]. Compared to the bulk OHPs, low-dimensional OHPs show obvious distinctions in their optical and electronic properties due to size-dependent effects [22,23,24]. Reported OHP nanostructures show strong PL emissions, especially for colloidal quantum dots (QDs) with diameters less than 10 nm [25]. Early demonstrations of a perovskites-based LED using polycrystalline films suffer from low external quantum efficiency (EQE) due to the nonideal PL quantum yield (QY) of the bulk OHPs [26]. Therefore, low-dimensional OHPs present potential alternatives for efficient and bright light-emitting devices [7,23,27,28,29,30,31,32,33]. Furthermore, nanowire and nanoplatelet-shaped OHPs open up a new class of materials for laser investigations [13,14,15,16]. Understanding the scale effects in OHPs is of great importance to improve the device performance and develop other functional devices.

**Figure 1:**
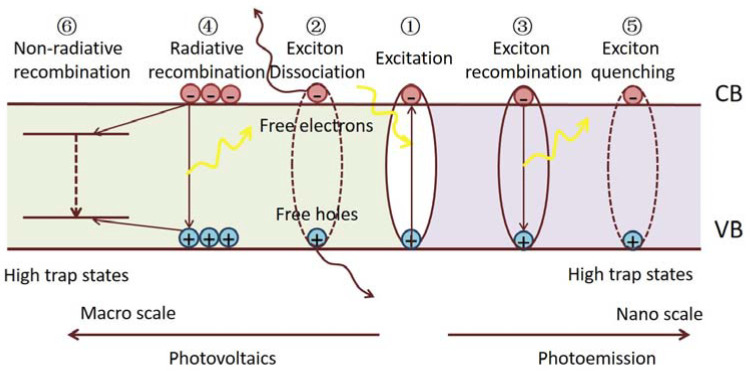
Schematic diagrams of the excited state features and the following exciton/carrier behaviors in OHP materials with scale dependency.

Generally speaking, the scale of OHPs strongly influences the optical and electronic properties correlated with their photo-exited properties and defect characteristics. Figure 1 depicts the features of excited states and some fundamental exciton/carrier processes in OHPs. Upon excitation, hot carriers relax on a sub-picosecond time scale to excitons (Process 1) [34]. The excitons typically separate into free carriers. Otherwise, they recombine and re-form the ground state. Aside from expected temperature dependences, it is evident that the physical scale of the material primarily decides the probabilities of these exciton dynamics in the hybrid perovskites. We give some examples below.

In bulk perovskite samples, excitons efficiently dissociate into highly mobile free electrons and holes within 2 ps, whereas the fraction of the remaining bound excitons is negligible (Process 2) [35]. The recombination of the free carriers depends on the defect number and energies [36] in the material. Photoluminescence (PL) analysis shows that the free carriers can recombine radiatively (Process 4) or by trap-assisted non-radiative recombination (Process 6). It is found that the intrinsic defects in OHPs can only form shallow levels near the band edges, whereas the surface/interface defects introduce trap states below the optical gaps and are associated with non-radiative recombination [37,38]. Remarkably, it has been discovered that even when using low-cost solution-based fabrication methods, polycrystalline films show long carrier diffusion lengths that exceed many state-of-the-art inorganic and organic semiconductors [39]. Furthermore, single-crystal OPHs with much fewer interface defects reveal extremely long charge carrier diffusion lengths of tens of microns [28]. The long diffusion length of bulk OHPs results from their high-charge carrier mobility and long carrier lifetime, which can be further ascribed to low bimolecular recombination rates in these materials. The small exciton binding energy, and hence efficient dissociation in bulk OHPs, long carrier diffusion length, and an absorption range covering the visible solar spectrum collectively enable the high performance of photo-electricity conversion.

On the other hand, by controlling the morphological dimensionality in low-dimensional structures such as two-dimensional (2D) quantum wells, one-dimensional (1D) nanowires, and zero-dimensional (0D) QDs, the excitonic absorption of these materials are largely enhanced [40,41]. Spectroscopic studies suggest that the PL emission comes mainly from the direct radiative recombination of photo-generated excitons (Process 3), which ensures a high PL QY [25]. The strong excitonic feature of low-dimensional OHPs and their tunable emission wavelengths make them promising emitters in LED and laser operations. It should also be noted that the defects, especially the surface defects, can cause non-radiative exciton recombination (Process 5), thus diminishing the PL efficiency.

In this progress report, we summarize some recent developments illustrating the scale-dependent effects of the electronic and photo-excited properties in OHPs. We will briefly introduce the synthesis of OHP materials at different scales from the morphological dimensionality and the molecular level. Then, we will specifically focus on the scale dependence of carrier diffusion, excitonic properties, and defect effects in these OHPs.

## 2. Crystal Structure of Organometal Halide Perovskite

Organic–inorganic hybrid perovskites can be expressed as (RNH_3_)_2_(CH_3_NH_3_)_n−1_B_n_X_3n+1_, where R = butyl, phenethyl, etc.; B = Pb, Sn; X = Cl, Br, I; *n* = 1, 2,…, ∞ [42,43]. Due to the large size of the organic cation, it usually forms a quantum well-like layered structure. The inorganic divalent cation B and halogen anion X form a set of corner-sharing BX_6_ octahedra layers, which is in-plane covalently bonded. In contrast, the organic cations can only link to the outer edge of the octahedra set. The inorganic and organic layers are stacked alternately and bonded by van der Waals force.

The structure of the OHP would evolve from a less symmetric orthorhombic structure to a highly symmetric tetragonal and ultimately cubic structure if the size of the organic cations is reduced. This type of cubic structure perovskite adopts the general chemical formula of ABX_3_, where the BX_6_ octahedron is at the center of the cube with X located on six face centers, and organic cation A occupies the corner of the cubes. The crystal structure of perovskite is influenced by a tolerance factor *t*, which is associated with the radii of A, B, and X:

(1) $$t=\frac{R_{A}+{\;R}_{X}}{\sqrt{2}\left(R_{B}+R_{X}\right)}\;$$

where *R*_A_, *R*_B_, and *R*_X_ are radii of A, B, and X. A stable cubic structure perovskite is likely to form when 0.89 < *t* < 1.0.

**Figure 2:**
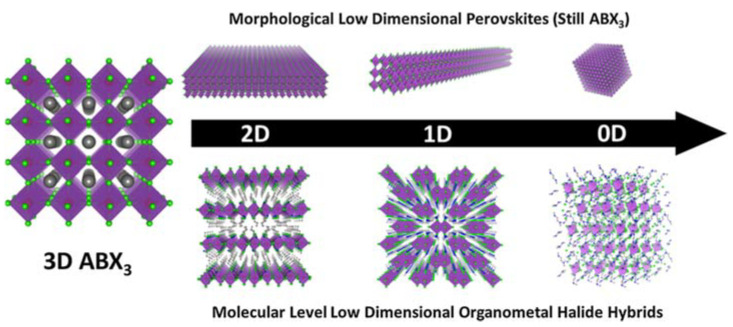
Three-dimensional ABX_3_ metal halide perovskites and perovskite-related materials have different morphological and molecular dimensionalities. [44] Copyright 2018, Elsevier B.V.

Low-dimensional perovskites can be generally classified into two distinct categories: morphological low-dimensional perovskites and molecular-level low-dimensional perovskites. Morphological low-dimensional perovskites trace their origins to 3D perovskite structures endowed with quantum confinement effects. Within this category, we find 2D nanoplatelets, 1D nanorods, and 0D quantum dots. These low-dimensional perovskite materials share an identical molecular composition and crystal structure with their 3D counterparts, with their quantum confinement effects emanating from variations in material dimensions and morphology. Conversely, molecular-level low-dimensional perovskites materialize within bulk crystals, where metal halide species stand isolated from one another, courtesy of organic cations. This distinct class of materials has the capacity to manifest the distinctive properties of individual building blocks upon separation. Diverging from the characteristics of morphological low-dimensional perovskites, the properties of molecular-level low-dimensional perovskites remain impervious to crystal size fluctuations, and their molecular composition resists expression in terms of ABX_3_. Figure 2 serves as a visual exposition, elucidating the process of dimensionality reduction from 3D to 2D, 1D, and 0D, both in the morphological and molecular contexts, ultimately yielding novel materials associated with ABX_3_ metal halide perovskites.

## 3. Synthetic Techniques

A prerequisite for investigating OHPs’ scale effect is fabricating perovskite materials from a single crystal to low-dimensional nanostructures [45,46]. This topic is beyond the scope of this progress report to deal with this broad topic, but a brief description is given.

### 3.1. Single Crystals

A facile and versatile way to grow single-crystal perovskites is the cooling method. Typically, lead acetate (PbAc) is selected as the lead source and dissolved in hydriodic acid (HI) together with CH_3_NH_3_X (MAX, X = Cl, Br, I) at high temperatures to form a saturated solution. By gradually cooling down the solution to a lower temperature, perovskites begin to precipitate from the supersaturated solution and grow into single crystals [47]. An improved route is the top-seeded solution growth (TSSG) method [28]. A controlled temperature gradient from bottom to top in the precursor solution is realized, and single-crystalline perovskite grows on the seed at the cooler top. Another alternative approach to growing single-crystal perovskite is anti-solvent vapor-assisted crystallization (AVC), which utilizes various solutions’ solubility differences. Dichloromethane (DCM) [48] or toluene [49,50] is selected as an anti-solvent in which the perovskite precursors are entirely insoluble and the slow diffusion of the anti-solvent into the good solvent solution containing the precursors induces the crystallization and produces large single crystals. 

The Frank Liu research group has reported an effective additive strategy for the growth of 2-inch-sized hybrid cation mixed-halide perovskite (HCID). This strategy effectively reduces iodide oxidation and cation deprotonation, which are responsible for phase segregation. As a result, the fabricated perovskite photodetector, termed FAMACs SC, exhibits a remarkable, more than five-fold enhancement in carrier lifetimes, high-charge mobility, a long carrier diffusion distance, superior uniformity, and long-term stability. These improvements enable the design of high-performance, self-powered integrated circuit photodetectors. The device demonstrates large responsivity, high photoconductive gain, excellent detectivity, and a fast response speed. Notably, all these values are among the highest reported for planar-type single-crystalline perovskite photodetectors [20].

Although the above methods provide universal routes to grow the single-crystal perovskites, the procedure is time-consuming. It usually takes several days to grow macroscale single crystals. Bakr’s group invented an inverse temperature crystallization (ITC) method in which the time for the crystallization process is significantly reduced to several minutes [51,52]. Such a method is established on the fact that the methylammonium lead trihalide perovskites (MAPbX_3_) have inverse temperature solubility in specific solvents at elevated temperatures. Single-crystal perovskite can precipitate from the saturated stock solution in certain solvents by heating rather than cooling. This rapid growth method paves the way for the mass production and further advances of these remarkable materials.

### 3.2. Polycrystalline Films

OHP tends to form cuboid polycrystals through either solution processing or vapor deposition methods. The resulting polycrystalline thin films can be an active layer in a PV device. Therefore, these structures have been intensely investigated due to the booming works on perovskite solar cells. Generally, the solution-based fabrication of polycrystalline perovskite thin films can be divided into two categories: the one-step spin-coating method [53] and the two-step sequential deposition method [3,54]. For the one-step method, precursors are mixed and dissolved in a single solution and spin-coated on the target substrate, and then the crystallization process gets underway upon heating. In the two-step method, the precursors are separately dissolved; in most cases, inorganic metal halide is first deposited on the substrate by spin-coating. The second-step deposition of the organic halide precursor can be realized by either spin-coating or simply immersing the substrate into the precursor solution. Thermal crystallization by heating is usually needed after the deposition process.

Although the solution-processed fabrication is easy to operate, alternative methods are also investigated better to control the thin film’s quality and uniformity. Dual source vapor evaporation deposition provides a way to produce flat and uniform perovskite thin films [55,56]. However, the high-vacuum and high-temperature requirements for thermal evaporation inevitably increase the fabrication cost. A combination of the solution and the vapor-based deposition is called the sequential liquid–vapor phase deposition [57]. Inorganic lead halide is first deposited, similar to the solution-based method. Then, the resulting thin film is transformed into perovskite by reacting with the vapor of the organic halide precursor (e.g., MAI) in a chemical vapor deposition (CVD) oven. 

### 3.3. Morphological Low-Dimensional Perovskites

**Figure 3:**
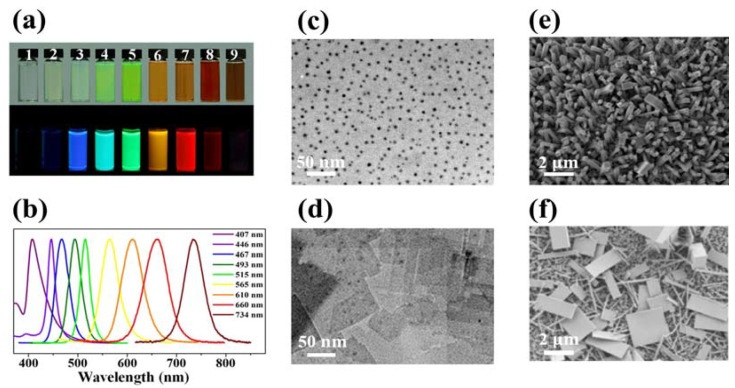
(**a**) Optical images of colloidal MAPbX_3_ (X = Br, I, Cl) QDs in toluene under ambient light and a 365 nm UV lamp. (**b**) PL emission spectra of MAPbX_3_ QDs. Reproduced with permission [25]. Copyright 2015, American Chemical Society. (**c**) TEM image of colloidal MAPbBr_3_ QDs. Reproduced with permission [61]. Copyright 2015, American Chemical Society. (**d**) TEM image of the general morphology of MAPbCl_3_ nanoplatelets. Reproduced with permission [60]. Copyright 2015, American Chemical Society. (**e**) SEM image of solution-processed MAPbI_3_ nanorod arrays. Reproduced with permission [66]. Copyright 2015, American Chemical Society. (**f**) SEM image of MAPbI_3_ nanoplatelets and nanowires. Reproduced with permission [67]. Copyright 2015, American Chemical Society.

There have been a number of studies on perovskite nanostructures such as 0D nanoparticles, 1D nanowires/rods, and 2D nanoplatelets/disks that possess enhanced optical properties that outperform their 3D bulk counterparts [46,58]. Instead of the hot-injection route for inorganic nanoparticles, ligand-assisted reprecipitation (LARP) synthesis adapted from organic nanoparticles and polymer dots is a general synthetic route for OHP colloidal nanoparticles [25]. Long-chain organic amines or their compounds are mainly selected as ancillary ligands. Precursors such as MAX and PbX_2_ (X = Cl, Br, I) are first dissolved in good solvent together with the long-chain organic molecules (e.g., octylammonium halide, octylamine, etc.). Then, the prepared solution is added dropwise into a bad solvent (e.g., toluene) to initiate the reprecipitation of the perovskite nanostructures. These colloidal QDs can be fabricated with tunable absorption and emission spectra through halide substitutions, as depicted in Figure 3a,b. Due to the lack of a suitable process to purify resulting QDs from the colloidal solution, an alternative approach named emulsion synthesis for OHP QDs is demonstrated. Precursors dissolved in a highly polar solvent (e.g., DMF) are dropped into a non-polar (e.g., hexane) [28,59] or weak-polar (e.g., 1-octadecene) [60] solvent together with a small addition of octylamine as ancillary ligands. Under vigorous stirring, a well-dispersed microemulsion is formed. Then, a third solvent, normally acetone, is introduced as a demulsifying agent, thus promoting the precipitation of colloidal perovskite nanoparticles (Figure 3c) [61]. Hassan et al. reported an alternative two-step method by reacting alky ammonium with as-prepared lead halide nanocrystals to synthesize perovskite QDs. The well-controlled monodispersed lead halide nanocrystal precursors are good templates for the growth and size control of the final products [62].

As discussed above, OHPs form essentially 2D quantum wells when prepared with large-size organic cations (Figure 3d). To obtain an atomically thin sample, a simple micromechanical exfoliation method from the bulk-layer structured perovskites is proposed [63,64]. The process is facile, but the resulting 2D nanosheets are in poor shape and uniform. The technique can only be applied to fundamental research and is unsuitable for quantity production. Alternatively, solution-based reprecipitation synthesis is also feasible for the mass synthesis of 2D-structured perovskite. A solution-based method to directly grow atomically thin 2D perovskite nanosheets is reported. The highly diluted precursor solution was dropped on Si/SiO_2_ substrates and dried. The spontaneous growth of atomically thin perovskite nanosheets is demonstrated as the solvent evaporated [65].

Besides the solution-based synthesis, vapor phase methods can also be employed for 2D perovskite. Xiong and co-workers reported a two-step CVD-processed synthesis of organolead halide perovskite nanoplatelets with thicknesses from several atomic layers to several hundred nanometers [35]. It is worth noting that by controlling the experimental conditions, 1D-structured perovskite nanowires can be obtained using such a method [16]. These vapor-phase-prepared low-dimensional single crystals with high crystallinity can be applied for high-quality nanolasers [13,16]. Particularly for the regular-shaped nanoplatelets, whispering-gallery nanolasers can be formed within the nanostructures with tunable wavelengths.

Besides vapor-phase synthesis, 1D-structured perovskite nanowires/nanorods are mainly synthesized via liquid-phase methods. The general experimental procedures are similar to the synthesis of perovskite polycrystalline thin films. Yang’s group reported a two-step solution-processed technique to fabricate MAPbBr_3_ nanorod arrays (Figure 3e) [66]. A surface-initiated solution growth strategy for the synthesis of high-quality single-crystal MAPbX_3_ (X = Cl, Br, I) nanowires, as well as nanoplatelets, is developed (Figure 3f) [14,67]. It is realized that keeping a low supersaturation condition through tuning the precursor concentration is crucial for successful nanostructure growth. The resulting 1D nanowires are also excellent templates for the use of lasers.

### 3.4. Molecular-Level Low-Dimensional Perovskites

Reducing the dimensionality of organic metal halide hybrids from 3D networks to 2D layers, 1D wires, and 0D molecular structures significantly reduces the conductivity in the direction in which the metal–halogen bonding network is destroyed. Self-trapping excitons (STEs) can be found in low-dimensional metal halogen hybrids [9,23,29,30,68,69]. By utilizing larger organic cations to isolate inorganic metal halides within crystals, a range of low-dimensional metal halide perovskites (LDMHPs) can be formed at the molecular level, encompassing two-dimensional (2D) layers, one-dimensional (1D) chains, and zero-dimensional (0D) clusters composed of octahedral metal halide units [24]. These LDMHP structures showcase notably distinct luminescent characteristics compared to their three-dimensional (3D) counterparts.

#### 3.4.1. Two-Dimensional Metal Halide Hybrids

**Figure 4:**
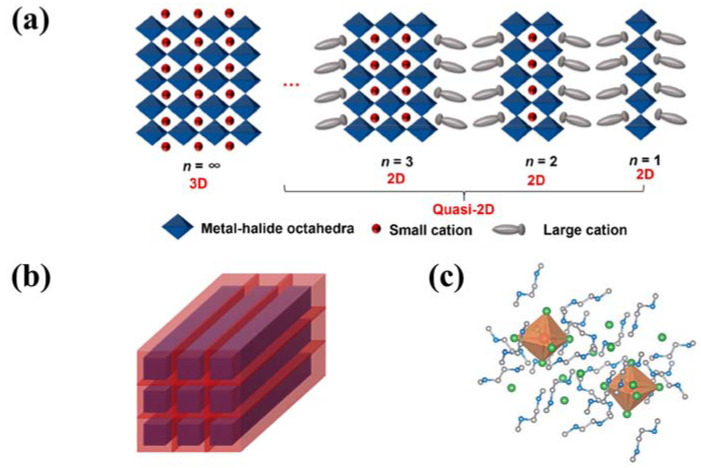
(**a**) Schematic representation of metal halide perovskites with 3D, 2D, and quasi-2D structures. Reproduced with permission [10]. Copyright 2020, American Chemical Society. (**b**) Schematic illustration of molecular level 1D structure (blue: metal halide chains/wires, red: organic cations). Reproduced with permission [44]. Copyright 2018, Elsevier B.V. (**c**) Schematic illustration of molecular level 0D structure—Individual [SnBr_6_]^4−^octahedra surrounded by (C_4_N_2_H_14_Br)^+^organic ions. Reproduced with permission [44]. Copyright 2018, Elsevier B.V.

Two-dimensional and quasi-2D organic metal halide hybrids have attracted more and more attention in recent years due to their unique photoelectric properties and higher stability than 3D perovskites. The general formula of such materials is A_n−1_A’_2_B_n_X_3n+1_, where A’ is a monovalent large organic cation as the spacer between inorganic layers, A is a small cation incorporated into the inorganic framework, B is the bivalent metal cation, and X is the halogen ion. According to the different number of metal halide layers, it can be divided into monolayer 2D (*n* = 1) or quasi-2D (1 < *n* < ∞) metal halide hybrids (Figure 4a).

Quasi-2D organic metal halide hybrids are a bulk assembly of 2D metal halide nanosheets whose inorganic layer thickness is more significant than a single 2D metal halide. Ma and colleagues reported a series of quasi-2D organic metal halide hybrids with the chemical formula (RNH_3_)_2_(CH_3_NH_3_)_n_Pb_n_Br_3n+1_ with a tunable emission spectrum ranging from deep blue to bright green [26]. Li and coworkers synthesized a halide quasi-2D PEA_2_MA_3_Pb_4_Br_13_; the poling methods are used in modulating the phase arrangement in quasi-2D perovskite LEDs [44].

#### 3.4.2. Three-Dimensional Metal Halide Hybrids

In 1D organic metal halide hybrids, the metal halide octahedron MX_6_ can form chains through corner-sharing, edge-sharing, or face-sharing. These anionic metal halide chains and the surrounding organic cations can be assembled to form a single-crystal structure. Unlike morphologically 1D metal halide perovskites, which are nanomaterials, 1D organic metal halide hybrids are indeed large crystals with a 1D structure at the molecular level [31] and can be considered to be bulk assemblies of metal halide quantum wires (Figure 4b). Lin and coworkers reported a novel molecular 1D organic-metal halide hybrid, [C_4_N_2_H_12_]_3_[PbBr_5_]_2_·4DMSO, in which the lead halide [PbBr_5_]∞ 1D chains, [C_4_N_2_H_12_]^2+^ organic cations, and DMSO molecules are assembled into single crystals. This material exhibits a warm white-light emission with an outstanding PLQE of 60% [32]. Ma and coworkers reported a novel 1D organic-metal halide hybrid (C_8_H_28_N_5_Pb_3_Cl_11_) consisting of metal halide nanoribbons with a width of three octahedral units. One-dimensional C_8_H_28_N_5_Pb_3_Cl_11_ is found to exhibit a dual emission with a high-energy emission peaked at 420 nm and a low-energy broad emission peaked at 540 nm due to the coexistence of free excitons and self-trapped excitons [70].

#### 3.4.3. Zero-Dimensional Metal Halide Hybrids

Ma’s research group reported a series of lead-free organic metal halide hybrids with a 0D structure, including (C_4_N_2_H_14_X)_4_SnX_6_ (X = Br, I) and (C_9_NH_20_)_2_SbX_5_ (X = Cl). The metal halide species were periodically doped in the wide band gap matrix. As a result of the excited-state structural reorganization of the individual metal halide species, highly luminescent strongly Stokes-shifted broadband emissions with photoluminescence quantum efficiencies (PLQEs) close to unity were achieved. The discovery of highly luminescent single-crystalline 0D organic–inorganic hybrid materials as perfect host–guest systems opens up a new paradigm in functional materials design [23]. Lin and coworkers prepared 0D H_3_SbBr_6_(L)_6_ [L = 2-(3-methyl-1*H*-imidazol-3-ium-1-yl)acetate] by a solvothermal reaction, which exhibits a greenish-yellow broadband emission with a PLQE of 53% [33]. Due to the environmentally friendly and high-stability features of tin (IV), 0D tin(IV)-based OMHHs have been developed and studied. For instance, 0D (C_6_N_2_H_16_Cl)_2_SnCl_6_ with a blue emission, which peaked at 450 nm, exhibited remarkable stability in air and at high temperatures [71]. Ma and coworkers reported using mechanochemical synthesis to prepare ionically bonded organic metal halide hybrids with a 0D structure at the molecular level. (Ph_4_P)_2_SbCl_5_ and (Ph_4_P)_2_MnCl_4_ were synthesized by grinding appropriate ratios of organic halide salt Ph_4_PCl with inorganic metal halide salts SbCl_3_ and MnCl_2_, respectively. Mechanochemical synthesis confers numerous benefits over solution-based processes, encompassing simplicity, cost-effectiveness, and environmental compatibility. It circumvents high-temperature reactions and minimizes solvent engagement. In specific instances, the inclusion of a few drops of solvents can induce the so-called liquid-assisted mechanochemical synthesis. Furthermore, recent investigations have demonstrated that mechanochemical synthesis can yield superior material properties and device performance in select cases compared to those obtained via traditional wet-chemistry synthesis [72].

### 3.5. Device-Oriented Synthesis

A consensus is that polycrystalline OHP thin film with a larger grain size performs better in PV device demonstration due to a reduced trap-state density with fewer grain boundaries [73,74,75]. Efforts have been made to increase the grain size [76,77,78,79]. By carefully controlling the MAI concentration and the exposure time of the first deposited PbI_2_ to the post-covered MAI solution before spin coating, MAPbI_3_ cuboids can be fabricated with a controlled size [80]. By adopting a hot-casting technique, the grain size of OHP films was extended to millimeter-scale [76]. A non-wetting method was invented to fabricate perovskite thin films with a large grain size [77]. Unlike the traditional liquid-based fabrication method that necessarily involves the pretreatment of the substrate to assure a contact angle as small as possible for better film formation, the idea in this work is to grow the perovskite on non-wetting hole transport layers (HTLs) to prevent densely formed nuclei from heterogeneous nucleation. Perovskite polycrystalline thin films with an average grain size/thickness aspect ratio of 2.3–7.9 are achieved when growing on non-wetting HTLs and show better structural and electrical properties such as fewer intrinsic and surface traps and higher charge mobility. An enhanced grain size has also improved the photo-stability of wide-bandgap MAPbBr*_x_*I_3–*x*_ solar cells [78].

While a large grain size is a prerequisite for superior PV performance, the requirement for highly efficient electroluminescence (EL) applications is quite the contrary. A grain or nanocrystalline size as small as possible is preferred for the demonstration of EL devices, in which the radiative recombination of the excitons can be spatially restrained in each nanograin/nanoparticle with less non-radiative recombination at their interfaces [81]. Currently, there are four ways to achieve the nanocrystalline films toward efficient light-emitting devices: (i) Uniform MAPbBr_3_ nanograins with an average grain size of 99.7 nanometers in diameter are synthesized through a nanocrystal pinning (NCP) method [81]. The idea of this method is to drip a volatile nonpolar solvent onto the spinning layers of precursor solutions to wash out the good solvent and induce fast crystallization by reducing the solvent evaporation time, leading to the pinning of nanocrystals. (ii) A modified sol-gel process enables ultrasmooth perovskite thin films with low roughness and a small domain size [82]. (iii) A blend of nanocrystalline perovskites and dielectric polymers results in pin-hole-free thin films with enhanced EL efficiency [5,83,84]. Such composite films take advantages in terms of simple processing and good film formability. (iv) Fabricating EL devices using the spin-casting of preformed QDs stabilized with long-chain surfactants.

## 4. Scale-Dependent Effects in Organometal Halide Perovskites

The fast development of synthetic routes for OHPs with the required size and scale makes it possible to investigate the scale-dependent properties of these materials.

### 4.1. Scale Effects of Perovskites with Morphological Difference

In conventional perovskite materials (ABX_3_), we manipulate the macroscopic properties of perovskites by controlling their shape and dimension. At the nanoscale, we can access various low-dimensional perovskite materials, such as 2D perovskite nanoplatelets, 1D perovskite nanowires, and 0D perovskite quantum dots. In the first part, we will discuss the scale effect from two aspects: carrier diffusion and excitonic properties.

#### 4.1.1. Carriers Diffusion

Diffusion length is a crucial parameter that describes carrier diffusion and is vital in determining the effectiveness of perovskite optoelectronic devices. Particularly for PV devices, the longer the diffusion length, the higher the opportunity for the charges to reach the interfaces without recombination. In principle, the diffusion length ($L_{D}$) is defined as the square root of the product of the diffusion coefficient (*D*) and carrier lifetime (τ):

(2) $$L_{D}={\left(D\tau \right)}^{1/2}$$

Here, the diffusion coefficient (*D*) is related to the carrier mobility (μ) by the Einstein relation:

(3) $$D=\mu \frac{k_{B}T}{q}$$

where $k_{B}$, T, and q are the Boltzmann constant, absolute temperature, and elementary charge, respectively. 

Normally, the diffusion coefficient and carrier lifetime can be extracted from the results of transient spectroscopy, including transient absorption (TA) [85], time-resolved photoluminescence (TRPL) [39], and transient terahertz spectroscopy (TTS) [86] by adopting the diffusion models. Alternatively, electric measuring approaches such as dark current–voltage (I-V) [48], time-of-flight (ToF) [28], and the Hall effect (HE) [87] could also be applied for the determination of charge mobilities.

Single-crystal perovskites possess low trap states and no grain boundaries, which is beneficial for a longer diffusion length. An extremely long diffusion length above 175 μm was reported for single-crystal MAPbI3 [28]. The high LD value can be explained by the anomalously high carrier lifetime measured in this work. Here, carrier mobilities were obtained from dark I-V characteristics according to the standard space charge-limited current (SCLC) theory and verified by HE and ToF techniques. Transient photovoltaic (TPV) and impedance spectroscopy (IS) are used to measure the charge lifetime instead of TA or TRPL. A lifetime of over 80 μs is obtained through such methods, which is almost two orders of magnitude greater than the lifetimes probed by spectroscopic techniques in other reports. It is conjectured that the affection of contacts and the reabsorbing of photons from radiative recombination give rise to the prolonged carrier lifetime.

As a comparison, Shi et al. reported a diffusion length of about 8 μm at the best case (using the best value of the measured mobility and carrier lifetime) in single-crystalline MAPbI_3_ with charge mobilities of 2.5 cm^2^ V^−1^ s^−1^ for both electrons and holes and a carrier lifetime of ~1 μs [48]. Besides the different measurement methods in determining the charge carrier lifetimes, the contact layers for the mobility measurement were not identical, leading to a relatively smaller value of the diffusion length. Similarly, a ~10 μm diffusion length at best case was reported by Saidaminov et al. The diffusion length in single-crystal MAPbBr_3_ is also reported, ranging from 1.3 μm at worst case to ~17 μm at best case [51].

Although the exact value of the diffusion length in single-crystal perovskites is still indeterminate, it is well recognized that the largely inhibited trap states in the single-crystal perovskites give rise to the long diffusion length. The density of the traps (n*_t_*) is calculated using the onset trap-filled limit voltage (*V_TFL_*), which can be obtained from dark I-V traces. The relation between n_t_ and *V_TFL_* is expressed as:

(4) $$n_{t}={2\varepsilon \varepsilon_{0}V}_{TFL}/eL^{2}$$

where *e* is the electronic charge, *L* is the thickness of the crystal, and ε is the dielectric constant of the material.

The trap density in the single crystal is calculated to be 1.4–3.6 × 10^10^ cm^−3^ [28,48,51] in single-crystal MAPbI_3_, which is not only about five orders of magnitude lower than the value in polycrystalline perovskite, but also lower than a variety of state-of-the-art optoelectronic inorganic and organic semiconductors [88,89,90,91].

A striking feature of the OHP is that the polycrystalline thin films can be readily prepared using the low-cost liquid-phase method while the film quality is quite comparable to that using the high-cost vapor-phase method. Due to the sizeable trap-induced recombination rate in polycrystalline perovskite thin films, their diffusion lengths are expected to be much shorter than those in their single-crystal counterparts. Nevertheless, the carrier diffusion lengths in the OHPs of the polycrystalline phase are superior to many types of solution-processed materials. For instance, the carrier diffusion lengths in organic polymer devices fabricated by solution-processed methods such as spin-coating or chemical bath deposition are typically around 10 nm [92,93]. As the factors that affect the trap densities in polycrystalline perovskites, such as film thickness, film quality, and domain size, are too complicated to be standardized and quantified, the reported diffusion lengths in polycrystalline OHP thin films range from ~100 nm to several microns [39,47,85,86,94]. The diffusion lengths of electrons and holes were measured individually in MAPbI_3_ polycrystalline thin films by employing Phenyl-C_61_-butyric acid methyl ester (PCBM) as the electron transport layer and 2,2′,7,7′-tetrakis-(N,N-di-4-methoxyphenylamino)-9,9′-spirobifluorene (Spiro-OMeTAD) for the hole transport measurement [85]. Relatively small diffusion lengths of about 100 nm for electrons and holes were obtained, probably due to large trap states induced by an imperfect film quality. By improving the film quality using PbCl_2_ instead of PbI_2_ as a precursor in the fabrication process, a diffusion length over 1 μm was achieved for both carriers in the so-called I/Cl mixed halide perovskite MAPbI_3-x_Cl_x_ [39]. The diffusing balance between electrons and holes remains to be a controversial issue. Some reports provide the holes with a higher/lower diffusion coefficient than the electrons, leading to an unbalanced transport length between the two carriers [47,95,96]. It is observed that the diffusion length also increases with the film thickness [47]. A diffusion length of 6.3 μm for the holes is obtained with a 390 nm-thick film. In comparison, the diffusion length is only 0.46 μm when the film thickness is 95 nm. The abnormally high diffusion length of the single- and polycrystalline OHPs originates from the materials’ high carrier mobility and low bimolecular recombination rates. It reflects their unique exciton and defect properties, as described below.

Low-dimensional OHPs hold promises for the development of nanoscale optoelectronics. It is essential to understand their photophysical properties before we can successfully incorporate them into device applications. The charge carrier diffusion length is a crucial parameter to evaluate the quality of the low-dimensional perovskites, especially for the nanostructured perovskites with a micron size at certain dimensions, namely, 1D nanowires or 2D nanoplatelets. To quantify the carrier diffusion along the micron-scale dimension, the carrier lifetime can be readily obtained from TRPL as adopted for bulk materials, whereas traditional techniques lack maneuverability for measuring the charge mobility of an individual perovskite nanowire or nanoplate mainly due to the difficulty of the deposition of electrodes on nanostructured materials. Tian et al. reported a direct observation and quantitative characterization of the carrier diffusion process in individual single-crystal MAPbI_3_ and MAPbBr_3_ nanowires and nanoplatelets using time-resolved and PL-scanned imaging microscopy [97]. The concept is to extract the PL signal at a certain distance from the excitation site and plot the PL intensity as a function of the delay time. The diffusion coefficient is deduced using a two-dimensional diffusion model. A trick is subtracting the wave-guided component from the PL traces to obtain pure carrier diffusion-induced PL kinetics. Through this method, a diffusion length of 14.0 ± 5.1 μm for MAPbI_3_ and 6.0 ± 1.6 μm for MAPbBr_3_ is calculated, which is comparable to the reported diffusion length in perovskite single crystals. Further investigation showed that the unique 1D geometry, the long carrier diffusion lengths, and low non-radiative recombination rates’ nature of the single-crystal perovskite nanowires are advantageous in demonstrating nanowire lasers [14]. An outstanding lasing performance with the low lasing thresholds and high Q factors for nanowire lasers is realized.

#### 4.1.2. Excitonic Features

**Figure 5:**
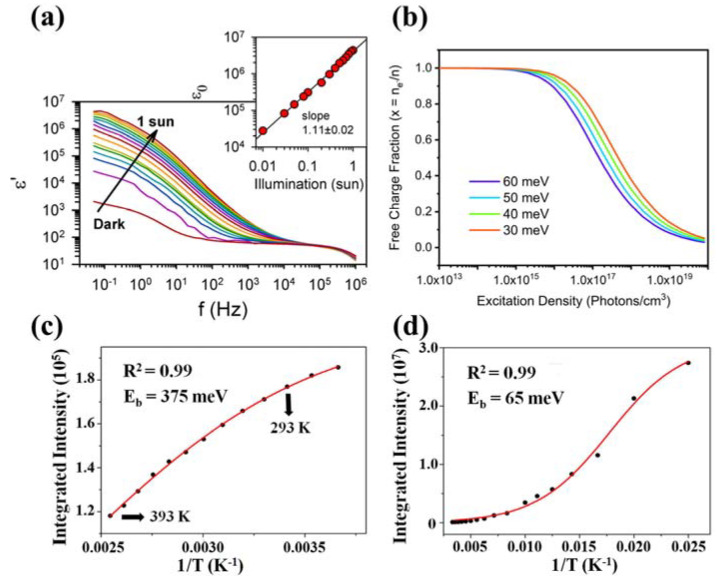
(**a**) The plot of the real permittivity as a function of frequency for different incident light intensities (Φ_0_) from dark to 1 sun for MAPbI_3−x_Cl_x_ perovskite. The inset shows the linear regression of dielectric constant vs illumination intensity at f = 50 mHz, observing a close-to-linear dependence between ε_0_ and intensity illumination. Reproduced with permission [102]. Copyright 2014, American Chemical Society. (**b**) Simulation of the free-charge fraction over the total excitation density at thermal equilibrium according to Equation (5), described in the text. Reproduced with permission [106]. Copyright 2023, the Owner Societies. (**c**) The plots of integrated PL emission intensity as a function of temperature of MAPbBr_3_ QDs (273–393 K) and (**d**) corresponding bulk material (50–300 K). Reproduced with permission [25] Copyright 2015, American Chemical Society.

An exciton is an electrically neutral quasiparticle defined as a bound state in which a photoexcited electron and a positively charged electron hole are bound to each other by Coulomb force. Depending on the binding energy of the electron–hole pairs, excitons can be categorized as Frenkel-type or Wannier-type. Excitons in 3D bulk perovskite MAPbX_3_ (X = Cl, Br, I) are treated as Wannier-type excitons with small exciton-binding energies, usually below 100 meV. For example, the exciton binding energy of hybrid perovskite MAPbI_3_ at room temperature is reported, ranging from a few meV to ~60 meV [86,98,99,100,101]. The relatively weak exciton binding energy can be explained by the substantial dielectric constant of the 3D bulk perovskite, which effectively screened the Coulomb interactions between the electron–hole pairs in the excitons. Bisquert and co-workers examined the dielectric constant of bulk perovskite MAPbI_3_ in the dark to be ~103 at low frequencies. Furthermore, they discovered a carrier density-dependent behavior of the measured dielectric permittivity [102]. A clear relation between the dielectric constant and the carrier density in the materials is observed by modifying the population of carriers with variations in illumination intensity, applied bias, or temperature. As depicted in Figure 5a, the dielectric constant rises to ~106 under the illumination of one sun. Huang’s group reported a slightly lower dark-state dielectric constant (still high enough for Coulomb screening) of 500 at low frequencies, mainly due to different material fabrication strategies [59].

Further investigations reveal that bound excitons can easily dissociate into free charges in bulk perovskites at room temperature. Transient absorption spectroscopy presents a direct means to observe the exciton and free-charge dynamics of perovskite materials [103]. Since the typical lifetime of the free charges is orders of magnitude longer than that of the excitons, the rapid decay component in the transient absorption traces can be assigned to exciton recombination, and the mild decay component is attributed to free-charge recombination. It is confirmed through this method that at relatively high temperatures (>250 K), free charges represent the dominant population (>94%) in the perovskites.

To sum up, for bulk single- or polycrystalline perovskites, the large dielectric constant screens the Coulomb attraction between carriers, ultimately leading to a small amount of exciton binding energy and facilitating rapid and efficient electron–hole separation. The fast exciton dissociation behavior at room temperature classes itself as a nonexcitonic material. The nonexcitonic nature of bulk OHPs partially explained their extraordinary performance in PV applications.

It is suggested that the large static dielectric constant, even in the dark, as well as spontaneous exciton dissociation is an intrinsic property of the organic–inorganic hybrid materials. Electronic structure analysis using density functional theory (DFT) shows that organic cations of OHPs play an important role in determining their electronic properties [104]. The free rotation of polar organic molecules at the high-temperature phase (cubic phase) ensures the fluctuation of the band structure of bulk OHPs; that is, the conduction band minimum (CBM) has dynamical positions around a high-symmetry point in the k-space, forming a basin that helps the rapid relaxation of the photoexcited electrons to the CBM with the assistance of acoustic phonons and separates the excitons into free carriers at the same time. The relaxation of the electrons to the slightly indirect CBM also results in a prolonged lifetime of the minority carriers, thus partially explaining the long diffusion lengths in the materials.

It is noted that halogen substitution influences the excitonic feature of the materials. For example, MAPbBr_3_ has a relatively larger bandgap and larger exciton binding energy of 80 meV, as compared to MAPbI_3,_ and distinct excitonic peaks of MAPbBr_3_ flat thin films at the band edge can be observed at room temperature, which is often attributed to the excitonic transition. Incident photon-to-current efficiency (IPCE) characterizations of the MAPbBr_3_ and MAPbBr_3-x_Cl_x_-based devices deposited by different electron transport layers were performed [59]. The IPCE intensity at the exciton peak of the Br or Br/Cl-mixed halide perovskite with respect to other wavelengths does not change with the variation of the applied bias, suggesting that the exciton dissociation process is not field-dependent. That is, despite the larger exciton binding energy by halogen substitution, the Br and Br/Cl-mixed halide perovskites should still be treated as nonexcitonic materials.

The exciton dissociation behavior also depends largely on temperature. Unlike the predominant population of free charges at high temperatures (>250 K), it is observed by temperature-dependent transient absorption spectroscopy that, at low temperatures (<160 K), the bulk MAPbI_3_ presents an orthorhombic structure and the recovery of the ground state bleaching is comprised of both free charges and excitons. The change in the ratio of free charges and initially created excitons, along with temperature variation, can be explained by the structural dependence of the dielectric constant. The ordered orientation of the organic cation group in the low-temperature orthorhombic phase results in a smaller dielectric constant, and thus a larger exciton binding energy [103]. At its extreme, sharp and strong excitonic peaks at the absorption edge can be observed for both MAPbI_3_ and MAPbBr_3_ at liquid helium temperatures (~5 K) [99,105].

If we denote *n* as the total density of excitation upon illumination, then ${n=n}_{FC}+n_{exc}$, where $n_{FC}$ and $n_{exc}$ is the density of free charges and bound excitons, respectively. *x* = $n_{FC}$/n is the fraction of free charges over the total density of excitations. The theoretical model that describes the relations between *x*, *n*, and temperature *T* for a 3D semiconductor can be expressed as [106]:

(5) $$\frac{x^{2}}{1-x}=\frac{1}{n}{\left(\frac{2\pi \mu k_{B}T}{h^{2}}\right)}^{3/2}e^{-\frac{E_{B}}{k_{B}T}}$$

where *E_B_* is the exciton binding energy, *m* is the reduced mass of the exciton (approximated to 0.15 m_e_), and *h* is Planck’s constant. By plotting *x* as a function of the total excitation density, *n*, and taking into account the reality of operating optoelectronic devices with *n* below 10^15^ cm^−3^, the dominance of the free-charges population is verified at around room temperature (Figure 5b), while the population of excitons starts to increase with a temperature reduction. These simulation results are well consistent with the experimental observations.

The excitonic properties of semiconductors dramatically change when the scale of the material goes to the nanosized dimension [107]. The high exciton binding energy is measured in QD-structured OHPs. Using temperature-dependent PL analysis, the exciton binding energy of ~375 meV is reported for MAPbBr_3_ QDs [25]. As a comparison, the bulk materials have an exciton binding energy of ~65 meV. The integrated PL evolution as a function of temperature for MAPbBr_3_ QDs and the bulk counterparts are presented in Figure 5c,d.

Distinct excitonic behavior can also be observed in 0D perovskite QDs with an average diameter under 10 nm, approaching the excitonic Bohr radius. Due to the small Bohr radius of the perovskite materials, the quantum size effect in QDs is usually in the weak confinement regime with a QD diameter larger than ~4 nm. It has been suspected that the reported quantum confinement effect of MAPbBr_3_ QDs with 6 nm in diameter in a previous work actually originated from the properties of the bulk perovskite phase [108]. The OHP QDs also showed a narrow band emission feature with FWHM of 20–50 nm and a small Stokes shift of ~40 meV, indicating that the PL emission of QDs mainly originates from direct exciton recombination.

**Figure 6:**
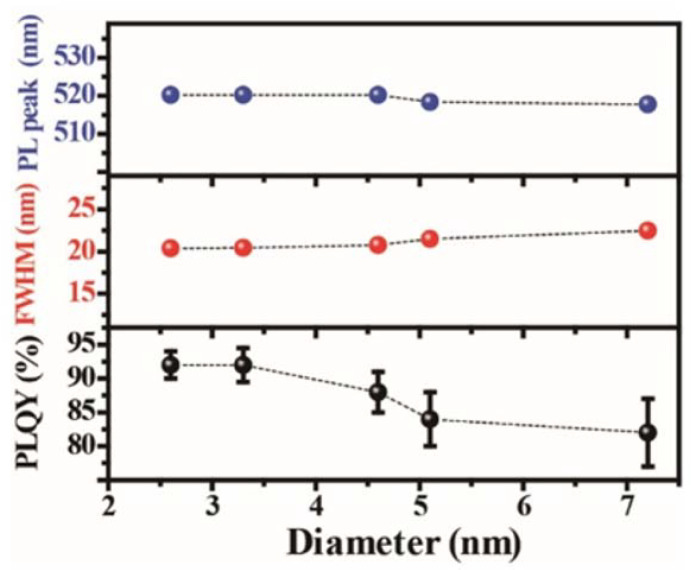
PL emission peak, FWHM, and PLQYs as a function of the size of MAPbBr_3_. Reproduced with permission [61]. Copyright 2015, American Chemical Society.

The monolayer perovskites show a high emission peak energy of 2.90 eV [109]. As the thickness increases, the emission peak red-shifted, illustrating the decrease in the bandgap. This confinement becomes weak after five layers. In perovskite QDs with an average size less than 2.8 nm, the peak energy decreases dramatically as the size increases [110]. Due to the small Bohr radius, the size dependence of the peak energy of the perovskite QDs is not so evident for the samples with larger average sizes ranging from 2.6 nm to 7.2 nm (Figure 6) [61]. This phenomenon of weak size-dependence in the regime above the Bohr radius, combined with the tunable emission peaks with halide substitution and the narrow FWHM feature, classify the perovskite QDs as a potential alternative for display technology. Furthermore, when the size of the nanocrystals is larger than 10 nm, the peak energy decreases gradually [111,112]. The MAPbBr_3_ single crystal shows an emission peak of 2.25 eV [19]. Like MAPbBr_3_, the bandgap of 2D layered MAPbI_3_ also strongly correlates with the layer thickness. For the layer number varied from 1 to 3, the bandgap decreases from 2.4 eV to 2.1 eV [38]. Quantum confinement effects of the MAPbI_3_ nanocrystals are also demonstrated [12,62,112,113]. As the crystal size increases, the bandgap gradually decreases and reaches 1.5 eV in the case of single crystals [51].

On the other hand, a few reports give the QY of the perovskite QDs with an average diameter of about 6 nm to be 17–20% [114,115]. The non-ideal QY is partially due to the large size of the QDs. By reducing the size of the QDs to an average of 3.3 nm in diameter, the QY of the MAPbBr_3_ QDs is enhanced to remarkably 50–70% [25]. The PL QY of MAPbBr_3_ QDs can be further improved to over 90% with their size reduced to 2.6 nm [28]. Besides the size reduction, proper chemical passivation on the QD surface also enhances photoemission efficiency. It is verified in the demonstration of the EL device that excess metallic lead atoms at the grain boundaries serve as nonradiative recombination sites and cause severe exciton quenching, thus damnifying the photoemission [81]. In the case of QDs, the grain boundaries become particle surfaces. It has proved effective in both cases that an appropriate increase of the bromide molar ratio can suppress the formation of metallic lead atoms at the grain boundaries/QD surfaces, thus reducing the surface/interface trapping effect. X-ray photoelectron spectroscopy (XPS) characterization shows the absence of metallic lead ion peaks in the Br-rich samples compared to the stoichiometric counterparts.

### 4.2. Scale Effects of Perovskites with Molecular-Level Difference

As discussed above, the basic building blocks of halide perovskites (metal halide octahedra, [BX_6_]_4_) can be arranged and connected in different ways to form three-dimensional (3D), two-dimensional (2D) and quasi-two-dimensional (2D), and one-dimensional (1D) structures, or they can be separated to form zero-dimensional (0D) crystalline structures [44]. Intrinsically, these structures are still composed of corner-sharing metal halide octahedra, and their crystallographic configurations remain identical to those of bulk 3D metal halide perovskites [44]. Notably, the properties of low-dimensional materials at the molecular level remain independent of their crystal size. Within bulk crystals, the individual metal halide species (layers, wires, or polyhedrons) are isolated from each other by organic cations. This isolation allows bulk crystals to manifest the intrinsic properties of the individual building blocks.

Each class of these materials exhibits distinctive physical and chemical properties, offering a diverse range of potential applications, notably in optoelectronic devices such as PV, LEDs, photodetectors, and lasers [24].

When the dimensions of OHP materials are further reduced to the quantum regime in all three dimensions, there still needs to be effective means to observe carrier diffusion. As described later in this article, it has been observed that the exciton binding energy is significantly enhanced in OHP quantum dots, indicating a predominant population of excitons rather than free carriers, as is the case in bulk materials. The spontaneous localization of excitons restricts their diffusion, so the exciton diffusion length in quantum dot films is expected to be much shorter. However, a reliable method for quantitative analysis is still under development.

Molecular excited-state structural reorganization is a well-established mechanism for explaining the significant Stokes shifts observed in many luminescent materials. Upon the absorption of photons, the metal halide species are elevated to high-energy excited states, which then rapidly undergo excited-state structural reorganization to transition to lower-energy excited states. This transition leads to broadband emission spectra with pronounced Stokes shifts and lifetimes measurable in microseconds. Similar below-gap broadband emission spectra have also been observed in corrugated 2D and 1D metal halide perovskites, attributed to exciton self-trapping. For metal halides, the formation of localized self-trapped excited states is highly dependent on the dimensionality of the crystalline systems, with reduced dimensionality facilitating exciton self-trapping [23]. Excitons in perovskites are self-trapped excited states that can have multiple trapped states with different energies depending on the ground-state electronic structure of the material. The formation of self-trapped excited states is critically dependent on the dimensionality of the crystalline systems, where lower dimensionality facilitates the formation of self-trapped excitons. In 1D and 0D perovskites, the excitons are confined to one or zero dimensions, resulting in strong quantum confinement and site isolation. In 2D perovskites, the excitons are confined to two dimensions, leading to anisotropic carrier transport and improved stability. In 3D perovskites, the excitons are free to move in all three dimensions, resulting in high carrier densities and efficient light absorption.

#### 4.2.1. Two-Dimensional and Quasi-Two-Dimensional Metal Halide Hybrids

A 2D layered perovskite can be prepared with the assistance of a large organic ligand. The naturally formed quantum well (QW) structure can be a prototype for investigating the exciton behaviors in the quantum-confined regions. The structure of 2D perovskites involves a periodic arrangement of inorganic layers, creating an analogy with multiple quantum well heterostructures. In both cases, there is a presence of strongly bound excitons. However, it is important to note that 2D perovskites do not exhibit the behavior characteristic of true quantum wells. Instead, the implications of this quantum-well-like structure are primarily observed in the excitonic behavior of layered lead halide perovskites [116].

The modulation of exciton binding energies in perovskite materials, particularly in the context of 2D perovskites, can be attributed to the interplay of quantum and dielectric confinement effects. Quantum confinement, as observed in 2D perovskites, results in a remarkable four-fold enhancement of the exciton binding energy (Eb) compared to its three-dimensional counterparts [116]. This enhancement stems from the reduced dimensionality of the material, which confines the excitonic wave functions more effectively. Dielectric confinement, on the other hand, arises due to the relatively weaker screening effect exerted by the organic layers within these structures. These organic layers exhibit a lower polarizability than the inorganic layers, reducing the Coulombic attraction between the electrons and holes [117]. Generally, as the number of the 2D perovskite layers decreases, the electric field lines of the Coulomb force between the excitonic electron–hole pairs are leaked outside of the perovskite layers. Since the dielectric constant of the long organic ligands surrounding the perovskite layers is much smaller than the perovskite itself [118], it reduces the Coulomb screening, thus increasing the exciton binding energy. A great enhancement of the exciton binding energy in the case of only a few perovskite layers is expected due to the minimal screening effect. The exciton binding energies for *n* = 1, 2, and 3 of layer-structured (C_4_H_9_NH_3_I)_2_(CH _3_NH_3_I)_n−1_(PbI_2_)_n_ are 360, 260, and 150 meV, respectively, which is an order of magnitude larger than the binding energy in the 3D bulk materials [45].

Hemamala I. Karunadasa’s research group comprehensively analyzed the exciton binding energies and band gap properties from multiple scholarly sources. Their findings revealed that perovskite compounds with values of “*n*” falling within the range of 1 < *n* < ∞ display intermediate characteristics lying between those exhibited by *n* = 1 and *n* = ∞ perovskites. For instance, within the (C_6_H_5_(CH_2_)_2_NH_3_)_2_(CH_3_NH_3_)_n−1_-Pb_n_I_3n+1_ family, the *n* = 1 member demonstrated energy values for the band gap (Eg) and the Eb at 2.58 and 0.220 eV, respectively. Similarly, the *n* = 2 perovskite exhibited Eg and Eb values of 2.34 and 0.170 eV, respectively. Notably, these energy parameters decreased further as “*n*” increased to *n* = 3. This observed trend in the exciton behavior was consistently observed in higher-*n* members of the (C_6_H_5_(CH_2_)_2_NH_3_)_2_(CH_3_NH_3_)_n−1_ series, resulting in a systematic redshift of luminescence as a function of the increasing “*n*”. Ultimately, this redshift converged towards the emission energy characteristic of the *n* = ∞ perovskite, denoted as (CH_3_NH_3_)PbI_3_, which manifested at 1.60 eV [44].

**Figure 7:**
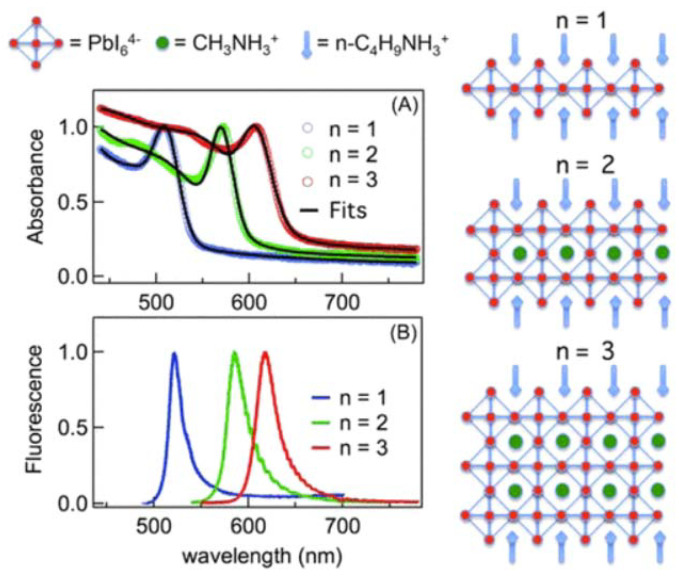
Room temperature absorption (upper) (**A**) and PL (lower) (**B**) spectra of 2D lead halide perovskite quantum wells: (C_4_H_9_NH_3_I)_2_(CH_3_NH_3_I)_n−1_(PbI_2_)_n_, *n* = 1, 2, 3. The right margin shows the structures of the samples, with annotations shown in the top margin. Reproduced with permission [34]. Copyright 2015, American Chemical Society.

The direct evidence of the enhanced excitonic features in 2D layered perovskites is the observation of distinct excitonic absorption peaks even at room temperature. Moreover, the decrease of the layered perovskites’ thickness down to the quantum confined regions also induces a blue-shift of their absorption onset and PL emissions. A sharp excitonic absorption feature of 2D layered MAPbBr_3_ with a single-unit cell thickness was observed, with 0.5 eV blue-shifted from that of its 3D bulk counterparts [108]. The absorption onset of the 2D MAPbBr_3_ continued shifting toward shorter wavelengths with more and more apparent excitonic absorption peaks and a decreased sample thickness, and monotonic blue-shift was also observed in PL-emitting bands (Figure 7) [109]. Furthermore, compared to the 3D counterparts, the Stokes shift in the 2D materials was smaller, indicating a dominated exciton recombination process. Quantum confinement effects depend on the relationship between the width of the quantum well, *l*, and the excitonic Bohr radius, *a*_B_ [119]. Two regimes can be defined by comparing the quantum well width to the Bohr radius. A strong confinement regime is defined as *l* ≪ $a_{B}$, while for *l* ≫ $a_{B}$, weak confinement is given.

For low-dimensional OHPs, the excitonic Bohr radius is around 1.36–2.0 nm [99,109,120]. The thicknesses of the 2D layered organolead lead perovskite, (C_4_H_9_NH_3_I)_2_(CH_3_NH_3_I)_n−1_(PbI_2_)_n_, were estimated from the thickness of the PbI_6_ octahedral layer to be 6.36, 13.7, and 19.1 Å for layer numbers *n* = 1, 2, and 3, respectively [34]. Therefore, the excitonic Bohr radius is comparable to the quantum well width with *n* below 3. Similarly, in the 2D layered organolead bromide perovskite, 2D nanoplatelets with *n* = 1 and *n* = 2 in thickness are assigned to be in the strong quantum confinement site isolation [24] regime and the samples with thickness *n* ≥ 3 are in the weak quantum confinement regime. It is observed that the electronic structure and property of the thick 2D perovskites (*n* ≥ 3) become more and more alike to that of the 3D bulk counterpart [109]. The thicker quantum wells (*n* = 3), consisting of three lead iodide octahedral layers, are less spatially deformable due to their more rigid structure than those in *n* = 1 and 2. Consequently, it is found that the excitons are less localized in the weak quantum confinement regime (*n* ≥ 3) than those in the strong confinement regime (*n* = 1, 2). The localized excitons in the 2D perovskite quantum wells give rise to the enhanced PL emission due to the radiative recombination of excitons becoming more effective. 

**Figure 8:**
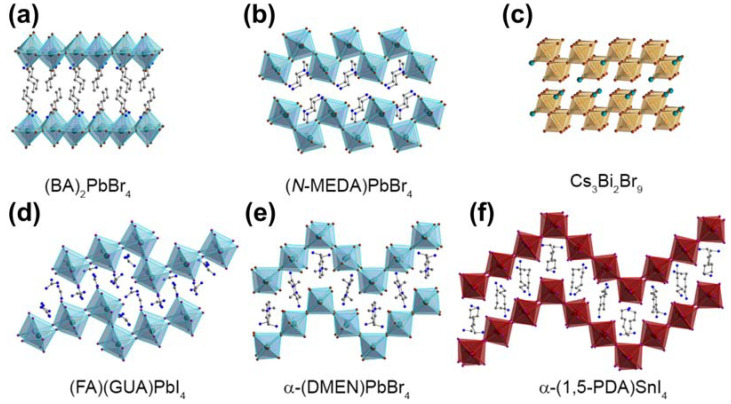
(**a**) Two-dimensional perovskite with planer structure (001). (**b**,**c**) Two-dimensional perovskite with simple corrugated structure. (**d**–**f**) Two-dimensional perovskite with complex corrugated structure [116]. Copyright 2019 American Chemical Society.

Two-dimensional halide perovskites can be conceptually envisioned as layers derived from their 3D parent structures, commonly called the Ruddlesden–Popper (RP) phase and can be cleaved along various crystallographic directions [121]. The general chemical formulation for RP-phase layered perovskites is denoted as L_2_A_n−1_B_n_X_3n+1_. In this formulation, ‘L’ means large cations, often encompassing large-sized or long-chain organic cations like butyl ammonium (C_4_H_9_NH_3_^+^ [BA^+^]). ‘A’ represents regular cations such as Cs^+^ or MA^+^, ‘B’ corresponds to divalent metal cations like Pb^2+^ or Sn^2+^, and ‘X’ signifies halide ions. The variable ‘*n*’ is an integer, indicating the number of metal halide octahedral layers situated between the two layers of L cations. These various crystal directions yield distinct planar structures, giving rise to various size effects. Different planar corrugations result in changes in the optical properties of perovskite materials [116]. One noteworthy category among these is the corrugated 2D halide perovskite, distinguished by its distinctive structural attributes and the concomitant size effects it manifests. As reported by the research group of Hemamala I. Karunadasa, materials with different corrugation structures can be assembled through solution-state self-assembly by replacing the A-site cations [116]. Figure 8 presents various planar morphologies of 2D perovskites under different compositions. Corrugated 2D layered perovskites are obtained by cleaving 3D perovskites along the (110) crystallographic direction [44]. In recent years, several research groups, including Mitzi et al., have synthesized a series of corrugated 2D layered perovskites with structures such as 2 × 2 and 4 × 4 [44]. Additionally, a range of corrugated 2D perovskites has been discovered recently, such as (N-MEDA)PbBr_4_ and (EDBE)PbX_4_. Mechanistic studies have indicated that exciton self-trapping results in emissions with a broad Stokes shift, while interactions between self-trapped excitons and permanent lattice defects lead to heterogeneity in the excited state, further contributing to emission broadening [116]. Mercouri G. Kanatzidis’ research has concurrently demonstrated a significant correlation between the bandwidth of PL emission spectra and the degree of distortion in corrugated 2D perovskites. The most severely distorted 2D perovskites exhibit the broadest emission bandwidth and the longest lifetime. The variation in the morphology of 2D perovskites similarly illustrates the size effects [122].

The highly efficient self-trapping mechanism in corrugated 2D structures results in broadband intrinsic white-light emission. In corrugated 2D layered perovskites, strong exciton–lattice coupling can induce transient lattice distortions, resulting in strongly Stokes-shifted broadband emissions [68], and these materials can simultaneously exhibit emissions from both free excitons and self-trapped excitons [44]. According to reports by Bi Wuma, the white light emitted by corrugated 2D structures can be tuned from “cool” to “warm” white light by varying the organic cations and halogen composition. However, achieving materials with high PLQEs remains a challenge.

#### 4.2.2. One-Dimensional Metal Halide Hybrids

Due to exciton self-trapping, corrugated 2D and 1D perovskites exhibit broad emission spectra. In 1D perovskites, the metal halide octahedra exhibit corner-sharing, edge-sharing, or face-sharing interactions, forming a 1D nanowire structure encapsulated by organic cations EDS. The structural arrangements within these nanowires can be either linear or zigzag, and their specific chemical compositions vary according to the bonding modes and the selection of organic cations. At the molecular level, 1D perovskites consist of numerous anionic metal halide chains isolated from each other and enclosed by organic cations. The distinct connectivity among the metal halide building blocks creates a unique electronic band structure. When considered as bulk assemblies of quantum wires, macroscopic crystals composed of molecular 1D perovskites exhibit properties identical to those of individual nanowires, irrespective of the crystal size [24].

#### 4.2.3. Zero-Dimensional Metal Halide Hybrids

In 0D perovskites at the molecular level, metal halide species are spatially encapsulated and isolated by wide-bandgap organic cations, forming highly localized excitons upon excitation. The origins of such highly localized excitations can be attributed to two main factors: firstly, the introduction of the dielectric confinement effect due to the small dielectric constant of the organic cations, and secondly, the quantum confinement effect arising from the minimal overlap of the wavefunctions of the metal halide species. Within 0D Organic–Inorganic Metal Halide Hybrids (0D OMHHs), the excitation and relaxation processes of excitons occur on individual metal halide species. Consequently, the valence band maximum and conduction band minimum of these materials correspond to the highest occupied molecular orbital (HOMO) and lowest unoccupied molecular orbital (LUMO) of the metal halide anions [71]. The interaction between excitons and phonons in 0D OMHHs results in the formation of self-trapped excitons (STEs). This process involves the structural reorganization of the metal halides in their excited state, leading to the formation of the lowest excited states, resulting in emissions with significant Stokes shifts [33].

The distinct morphology of 0D perovskites can also give rise to size-dependent effects. Studies on tin bromide and other 0D materials have uncovered a significant attribute of seesaw-shaped metal halides: their capability to exhibit highly Stokes-shifted broadband deep-red emissions. This distinctive property presents substantial potential for a range of applications in optoelectronics and sensing, thereby augmenting the prospects of these materials in pertinent technological domains [30,71].

### 4.3. Defect Effects

#### 4.3.1. Defect Effects in 3D Bulk Perovskites

As mentioned above, the electronic structure calculations of the rotational organic cations in OHPs show that an enhanced carrier lifetime can be ascribed to the relaxation of the electrons to the dynamical position of the CBM, which provides one possible reason for the long carrier diffusion length. On the other hand, defects in crystal materials generally act as recombination sites and are detrimental to carrier diffusion. Therefore, the role of defects in the OHPs is also worth investigating to understand the origin of a long carrier diffusion length from another perspective. Two categories of defects are of interest in 3D bulk OHPs: intrinsic and surface/interface defects. Two types of intrinsic defects are considered: the neutral vacancy pair defects (Schottky defects) and the elementary vacancy defects (Frenkel defects). Electronic structure calculations using DFT on the two groups of intrinsic defects in MAPbI_3_ were performed [37]. For Schottky defects, such as PbI_2_ and MAI vacancies, the formation energy of the PbI_2_ vacancy is relatively low (27–77 meV) compared with that of other semiconductors, while the formation energy of the MAI vacancy is relatively higher (1.803 eV). The low defect formation energy suggests the generation of abundant Schottky defects during the synthesis of bulk OHPs. Nevertheless, the density of states (DOS) of Schottky defects manifests that this type of defect does not make gap states within the band gap, which is quite exceptional compared to conventional semiconductors such as Si, group III-V, and group II-VI semiconductors. The ionic bonding nature due to organic–inorganic hybridization in MAPbI_3_ is considered to be the cause of the absence of gap states. The strong antibonding coupling of Pb lone-pair *s* orbital and I *p* orbital and the high ionicity of MAPbI_3_ give rise to the unusual defect behavior [123].

On the other hand, for Frenkel defects such as the elemental vacancies of Pb, I, and MA, DOS shows that the anionic I vacancy forms shallow levels at the conduction band edge and can be a source of unintentional *n*-type doping, while cationic Pb and MA create shallow levels near the valence band and provide sources for *p*-type doping. Hence, MAPbI_3_ can be synthesized to be *n*-type, intrinsic, or *p*-type semiconductors by manipulating the atomic composition in the growth process. It is believed that small effective masses for both electrons and holes and a large dielectric constant are essential for intrinsic defects in MAPbI_3_ displaying extended state characteristics rather than acting as Shockley–Read–Hall nonradiative recombination centers. The calculations also show that the formation energies for the intrinsic defects to generate deep levels are higher than those of the shallow levels. Therefore, the long carrier lifetimes and diffusion lengths of OPHs can also be partially attributed to the defect properties in these materials, which also serve as prerequisites for high efficient PV applications.

Surface or interface defects have a more apparent influence on the excitonic properties of the materials compared to the intrinsic defects. The above discussion shows that appropriate chemical passivation can effectively reduce defect-induced non-radiative exciton recombination at the sample’s surfaces/interfaces. It is observed by ultraviolet photoemission spectroscopy (UPS) that an additional density of states exists above the valence band maxima (VBM), indicating the presence of abundant hole traps on the perovskite surfaces [38]. The same formalism holds for electronic traps below the CBM. Excitonic traps below the optical gaps can also be observed using TA measurements. The energetic positions of these trap states have a relatively broad distribution at around 100–400 meV below the band gap, suggesting that these traps originate from the self-trapping of band-edge excitons and can be further ascribed to electron–phonon coupling at surfaces or interfaces of OHPs [68]. Due to the presence of these excitonic traps, the faster recombination of the charge carriers at the surface or interface of the OHPs than that within the grains is recognized. Nevertheless, single-crystal OHPs with no interior grain boundaries face less influence of the surface defects. It is measured in a MAPbBr_3_ single crystal that the surface recombination velocity is about $3.4\times 10^{3}\;cm\;s^{-1}$, which is 2–3 orders of magnitude smaller than that in many semiconductors [50]. However, for the OHP polycrystals with a large number of grain boundaries, the influence of surface/interface trap states cannot be neglected. PL analysis of polycrystalline OHP thin films also revealed that at a low excitation power, the PL QY is restrained due to carrier trapping at the subgap states. With elevated excitation fluence, the traps are gradually filled, and the efficient radiative recombination of the photoexcited carriers becomes dominant, leading to an enhanced PL QY [36,124].

The higher trap density in polycrystalline OHPs directly limits the device performance in PV operations. By carefully examining the planar structured perovskite solar cells with various grain sizes ranging from 100 nm to 500 nm, it is found that all device parameters, including the short-circuit current (J_SC_), open-circuit voltage (V_OC_), and fill factor (FF), are improved with the increasing grain size. While the enhancement in JSC is attributed to thicker film, the improved V_OC_ and FF are due to the reduced trap density, larger grain size, and fewer grain boundaries [75]. It is also found that the effective carrier lifetime is determined by the surface recombination process at the grain boundaries. The non-radiative recombination rates increased dramatically with the grain size decrease, resulting in a lower effective lifetime [50]. The better PV performance of OHP solar cells employing MAPbI_3−x_Cl_x_ films produced from PbCl_2_ than MAPbI_3_ films from PbI_2,_ as outlined above, can also be ascribed to the different defect features between these two films [39]. It is noted that investigations have come to a consensus that MAPbI_3−x_Cl_x_ seems to possess the same structure and stoichiometry as MAPbI_3_ [118]. Based on the TA observations, a lower density of surface/interface trap states in PbCl_2_-produced MAPbI_3_ perovskites is obtained, giving rise to the former material’s better performance. A more in-depth experiment revealed the spatial PL decay differentiations within grains and on the grain boundaries at a micron-scale using confocal fluorescence microscopy combined with in situ scanning electron microscopy [73]. The first finding was that the PL intensities and lifetimes varied a lot within each grain in the same film. The darker grains possess a red-shifted (~2 nm) and slightly broader PL band than the brighter grains, leading to a less sharp band edge. The observation provides evidence for the nonuniform distribution of defect-induced shallow trapping levels in polycrystal OHP films. Compared to the grains, the PL intensity at the grain boundaries was even dimmer, and the PL lifetime was shorter, indicating faster nonradiative decay. Therefore, the grain boundaries can serve as more efficient nonradiative recombination sites, thus deteriorating the PV performance. To solve this problem, growing high-quality OHP thin films with larger grain sizes is a way out, as mentioned above. It is estimated that the grain size should be larger than 30 μm to eliminate the impact of surface recombination at the grain boundaries [50]. Chemical passivation also proved effective in remediating the nonradiative defects at the grain boundaries. A comparative study on the PL features of the films with or without pyridine vapor passivation showed increased PL brightness and decreased PL bandwidth after pyridine exposure, indicating a reduction in the shallow trap density through chemical passivation [73].

#### 4.3.2. Defect Effects in Low-Dimensional Perovskites

For the low-dimensional OHP nanostructures, considering the size effects, the number of intrinsic defects becomes minimal with the volume shirking and even disappears due to the autoexcluding effects [125]. However, surface defects are dominant in nanosized materials due to the large surface-to-volume ratio. Through excitation-power-dependent PL analysis, it is proved that a threshold exists in the PL spectra of nanostructured MAPbBr_3_, which reflects the trap-filling process at low excitation powers and demonstrates the existence of surface defects [126]. The trap state dynamics in colloidal MAPbBr_3_ QDs and the corresponding bulk crystals are particularly discussed by Zheng and co-workers [126]. It is declared that the QDs are semi-passivated by the capping ligands during the synthesis process, which is quite incomplete, as the surface trap density of the QDs is even larger than that of the bulk crystals. Nevertheless, the exciton quenching by the trap states in the QDs is actually lower. The trap-assisted non-radiative recombination rate is almost 100% for the bulk crystal and 70% for the QDs. The most possible explanation is that the localization of the excitons, as mentioned above, restrains the interaction of the excitons and surface trap states; while in the bulk materials, the highly mobile free carriers could easily reach the unfilled traps. Even so, the surface defects still have non-negligible influences on the PL performances of the low-dimensional OHPs. In addition, in OHP nanostructures, especially for the QDs, the halide doping process for panchromatic light emission leads to compositional and structural variations, thus inevitably inducing more surface defects. Therefore, decreasing the density of surface defects is a priority to further enhance the photo- or electro-luminescent efficiency of the low-dimensional OHPs. As stated above, chemical passivation, such as surface Br-rich treatment, effectively reduces the surface defects. Without the influence of the surface traps, the excitons in the QDs can undergo nearly 100% radiative recombinations, which give rise to short PL lifetimes and high PL QYs.

#### 4.3.3. Recombination Kinetics

It is understood from the above discussion that in bulk OHPs, free carriers with long diffusion lengths occupy the dominant population under normal device operation conditions. More prominent factions of excitons are only observed at low temperatures or high excitation powers. Considering the intrinsic defects in OHPs form shallow levels while the surface and interface defects induce subgap trap states, comprehensive conclusions about the recombination dynamics in the bulk OHPs can be made.

**Figure 9:**
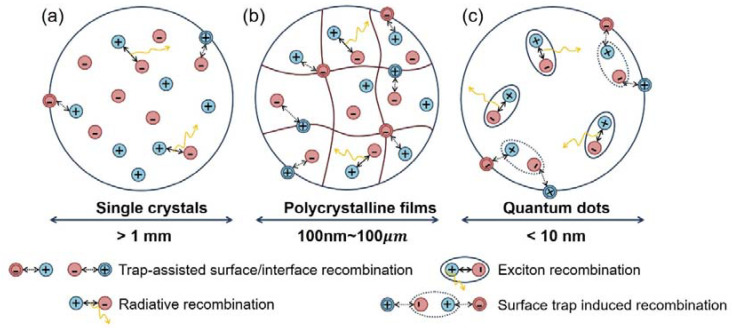
Illustration of recombination kinetics of OHP single crystals, polycrystalline films, and quantum dots, with annotations shown in the bottom margin. (**a**) Single crystals. (**b**) Polycrystalline films. (**c**) Quantum dots.

For bulk OHP single crystals at the macro scale, the influence of the surface becomes negligible. On the other hand, the absence of deep levels inside the sample ensures a high carrier lifetime and, thus, a long carrier diffusion length. Moreover, the long PL lifetimes [39] obtained from TRPL measurements confirm that the PL emissions mainly originate from the radiative recombination of free electrons and holes (Figure 9a).

In the case of polycrystalline OHP thin films, the influence of defects at the grain boundaries must be considered (Figure 9b). Surface/interface induced trap states diminish the PL QY at low excitation powers, and when such traps are predominantly filled at a high excitation density, a jump in the PL efficiency can be observed [36,124]. This phenomenon is also observed in low-dimensional OHP nanostructures [126]. Trap-assisted recombination at the grain boundaries is perceived as the primary cause that limits the performance of the OHP optoelectronics. In this regard, minimizing the effect of grain boundaries is of great significance in either PV or light emission applications. For solar cell operations, generally, two approaches can be applied: one is enlarging the size of the grains, and another is defect passivation at the grain boundaries. For EL devices, the grain sizes are fabricated as small as possible to confine the excitons, thus increasing the exciton recombination probability. This means the defect treatment at the grain boundaries to eliminate the trap-mediated non-radiative recombination becomes more urgent.

Excitons hold for the majority population in the low-dimensional OHPs, especially when the size of the materials goes down to the quantum confinement regime (Figure 9c). Hence, the recombination kinetics in OHP QDs are quite the opposite compared to their 3D bulk counterparts. These QDs can be treated as nanosized single crystals as there are no intrinsic defects inside the QDs on account of the size effects. The short PL lifetime and small Stokes shift [25] imply the dominance of direct exciton recombination. Due to the large surface-to-volume ratio, the non-radiative recombination of the excitons at the surface defects states are believed to be the primary cause of the reduced PL QY. Similar to polycrystalline OHPs, proper surface treatment is needed to alleviate the influence of surface defects. Notably, in a pertinent investigation by Biwu Ma, the tunability of OMHHs presents an avenue for manipulating the lattice softness by modifying the organic cations. This inherent tunability affords precise confinement regulation, facilitating comprehensive investigations into the intricate dynamics of excitons and charge carriers within the photoactive metal halide structures [71]. At the same time, some studies have pointed out the use of transient absorption spectroscopy to characterize the excited-state dynamics and kinetics of 1D and 0D perovskites. Transient absorption spectroscopy can be used to detect excited-state absorption and thus the time-resolved evolution of their population, which can help to better understand the exciton self-trapping (or excited-state structural reorganization) mechanisms in these low-dimensional perovskites [30].

## 5. Conclusions

The continual progress in the field of OHP-based photovoltaics has aroused intense interest in researchers with various backgrounds to explore the use of these materials beyond solar energy conversion. Since the requirements of materials in different optoelectronic devices vary a lot, it is of great significance to understand the fundamental chemical and physical properties of the OHPs, thus they can enlighten us on the future development trends of these materials. We here examined the scale dependence of crucial optical and electronic properties in these organic–inorganic hybrid materials mainly from three aspects: the carrier diffusion lengths, the exciton behaviors, and the defect states. The bulk OHPs show abnormally long carrier diffusion lengths even in the polycrystalline state, and the diffusion lengths become extremely long in macro-scale single crystals. Conspicuously, there is a large disparity between the excitonic features of 3D bulk and low-dimensional OHPs. Excitons are basically separated into free carriers in bulk OHPs. However, in low-dimensional OHPs, strongly enhanced exciton binding energy is observed, resulting in much more apparent PL behavior. From a theoretical point of view, the unique electronic and optical properties of OHPs are native features that stem from structural fluctuations of the organic–inorganic hybrid perovskite unit cell, which is also beneficial for a longer carrier lifetime. Intrinsic defects in the OHPs are proved insignificant in affecting the electronic properties due to their tendency to form only shallow levels in the band structure. Surface or interface defects are more detrimental to the device performance. It is believed that the grain boundaries in polycrystalline OHP thin film become nonradiative recombination sites for both PV and electroluminescent operations. For low-dimensional OHP structures such as nanoplatelets, nanowires, and QDs, a higher density of surface defects can definitely affect their properties, such as PL efficiencies.

The observations of size-dependent properties in OHPs remind us to take into consideration of the scale effects in designing the device structures for different applications. For instance, the development of high-efficiency OHP solar cells requires uniform OHP thin films with a grain size for which the larger is the better. Non-radiative trapping states should be carefully dealt with to further improve the carrier dissociation and transporting. While in the case of photo-electro-luminescent devices, a grain or particle size as small as possible is favorable as an enhanced quantum confinement effect can be realized to localize the excitons/carriers and strengthen the photoemission efficiency. On the other hand, more attention should be paid to the surface or interface defects during the fabrication, and proper chemical treatment is needed for the optimization of the device performances.

## Data Availability

Not applicable.
